# Serological evidence for historical and present-day exposure of North American bison to *Mycoplasma bovis*

**DOI:** 10.1186/s12917-020-02717-5

**Published:** 2021-01-07

**Authors:** Karen B. Register, Margaret Parker, Kelly A. Patyk, Steven J. Sweeney, William D. Boatwright, Lee C. Jones, Murray Woodbury, David L. Hunter, John Treanor, Marshall Kohr, Robert G. Hamilton, Todd K. Shury, Pauline Nol

**Affiliations:** 1 Ruminant Diseases and Immunology Research Unit, USDA/Agricultural Research Service/National Animal Disease Center, Ames, IA USA; 2 Center for Epidemiology and Animal Health, USDA:APHIS:Veterinary Services, Fort Collins, CO USA; 3 US Fish and Wildlife Service, Wildlife Health Office, Bozeman, MT USA; 4 Department of Large Animal Clinical Sciences, Western College of Veterinary Medicine, University of Saskatchewan, Saskatoon, SK Canada; 5 Turner Enterprises Inc., Bozeman, MT USA; 6 Yellowstone Center for Resources, Yellowstone National Park, WY USA; 7 Animal Medical Center of Wyoming, LLC, Gillette, WY USA; 8 The Nature Conservancy, Pawhuska, OK USA; 9 Parks Canada Agency, Saskatoon, SK Canada; 10 Wildlife Livestock Disease Investigations Team, USDA:APHIS:Veterinary Services, Fort Collins, CO USA; 11 Present address: Colorado Division of Parks and Wildlife, Wildlife Health Program, Fort Collins, CO USA

**Keywords:** *Mycoplasma bovis*, Bison, ELISA

## Abstract

**Background:**

*Mycoplasma bovis* causes mastitis, otitis, pneumonia and arthritis in cattle and is a major contributor to bovine respiratory disease complex. Around the year 2000, it emerged as a significant threat to the health of North American bison. Whether healthy bison are carriers of *M. bovis* and when they were first exposed is not known. To investigate these questions we used a commercially available ELISA that detects antibodies to *M. bovis* to test 3295 sera collected from 1984 through 2019 from bison in the United States and Canada.

**Results:**

We identified moderately to strongly seropositive bison from as long ago as the late 1980s. Average seroprevalence over the past 36 years is similar in the United States and Canada, but country-specific differences are evident when data are sorted by the era of collection. Seroprevalence in the United States during the pre-disease era (1999 and prior) was significantly higher than in Canada, but was significantly lower than in Canada during the years 2000–2019. Considering individual countries, seroprevalence in the United States since the year 2000 dropped significantly as compared to the years 1985–1999. In Canada the trend is reversed, with seroprevalence increasing significantly since the year 2000. ELISA scores for sera collected from free-ranging bison do not differ significantly from scores for sera from more intensively managed animals, regardless of the era in which they were collected. However, seroprevalence among intensively raised Canadian bison has nearly doubled since the year 2000 and average ELISA scores rose significantly.

**Conclusions:**

Our data provide the first evidence that North American bison were exposed to *M. bovis* many years prior to the emergence of *M. bovis*-related disease. Patterns of exposure inferred from these results differ in the United States and Canada, depending on the era under consideration. Our data further suggest that *M. bovis* may colonize healthy bison at a level sufficient to trigger antibody responses but without causing overt disease. These findings provide novel insights as to the history of *M. bovis* in bison and will be of value in formulating strategies to minimize the impact of mycoplasmosis on bison health and production.

## Background

The bacterial pathogen *Mycoplasma bovis* was first isolated roughly 60 years ago, from a dairy cattle herd in the United States experiencing a severe outbreak of mastitis [1]. Since that time it has spread gradually throughout the world and today has a significant negative impact on cattle health and production in nearly all countries where beef or dairy cattle are intensively raised as a food source [2, 3]. Clinical manifestations of disease are often chronic and include bronchopneumonia, mastitis, arthritis, otitis media, conjunctivitis and, more rarely, reproductive disorders [4]. Beginning around the year 2000, *M. bovis* additionally emerged as a health threat to North American bison. The first outbreak to be recognized occurred in the western United States in 1999, when ~ 20% of a group of yearlings on pasture died from pneumonia (D. Hunter, personal communication). The episode was attributed to *M. bovis* based on the presence of lung lesions resembling those of bovine mycoplasmosis from which *M. bovis* was the only microbial pathogen consistently identified. The next apparent outbreak occurred in 2001, when *M. bovis* was identified as the cause of severe pneumonia and arthritis in a Canadian bison herd [5]. In the years following, anecdotal evidence continued to accumulate further implicating *M. bovis* as a primary cause of disease in bison of all ages among herds in both the United States and Canada [5]. Subsequent studies firmly established its role as a primary etiologic agent of pneumonia, polyarthritis, necrotic pharyngitis, pleuritis, dystocia, and abortion in bison, with herd-level fatalities as high as ~ 30% [6–11].

An understanding of why *M. bovis* has emerged only relatively recently as a pathogen in bison would be of benefit in formulating control measures and strategies to minimize its impact on bison health and production. One hypothesis that has been proposed to account for this phenomenon theorizes that *M. bovis* has been circulating among North American bison for much of their history, or at least since the time it was first recognized in cattle, and that the appearance of mycoplasmosis in bison was triggered by intensified production and related changes in management and handling or by the rise of new genotypes with heightened virulence attributes [12]. An alternative theory posits that the first incursion of *M. bovis* into North American bison was more recent and was immediately followed by the appearance of *M. bovis*-related disease. No data currently exist that support or refute either of these possibilities. However, banked bison sera collected over the past 36 years provide a resource that can be used to investigate the historical and current prevalence of exposure to *M. bovis* in bison. The goal of our study was to determine whether there is serological evidence to suggest that *M. bovis* was circulating in bison well in advance of the time it was first recognized as a pathogen of bison (roughly the year 2000) and, if so, to compare historical and present-day rates of seroprevalence.

## Results

### Sera parsed by country of origin and/or era of collection

Collated ELISA results are provided in Table 1, with sera grouped by year of collection and individual location of origin. A total of 227 sera tested positively for antibodies to *M. bovis* (6.9% of the 3295 evaluated), with 126 weakly positive (1+; 3.8% of all sera, 55.5% of all positives) and 101 moderately (2+ or 3+) or strongly positive (4+ or 5+; 3.1% of all sera, 44.5% of all positives). The proportion of positive sera for samples acquired on a single occasion from a single source ranges from 0 to 66.7%. Seropositive bison were identified in 16/23 (69.6%) American herds, ranches or locations represented in this study and 16/18 (88.9%) Canadian sources.

**Table 1:** Summary of the origin, year of collection and ELISA score for the bison sera evaluated

Country and site of origin^a^	State or province of origin	Management	Year of	No. of	ELISA score					
		status	collection	sera	0	1+	2+/3+	4+/5+	% neg	% pos
Can-N	Northwest Territories	MM	1984–1987^b^	58	56	1	1	0	96.6	3.4
US-E	South Dakota	unknown	1985	8	8	0	0	0	100	0
US-D	Wyoming	unknown	1988	214	200	10	1	3	93.5	6.5
Can-P	Northwest Territories	MM	1994	126	120	4	2	0	95.2	4.8
Can-H	Alberta	RM	1996	65	63	2	0	0	96.9	3.1
Can-I	Alberta	RM	1996	41	39	2	0	0	95.1	4.9
Can-J	Alberta	RM	1996	46	46	0	0	0	100	0
Can-K	Alberta	RM	1997	89	87	1	1	0	97.8	2.2
Can-L	Alberta	RM	1997	47	43	1	3	0	91.5	8.5
Can-B	Alberta	RM	1997	12	11	0	1	0	91.7	8.3
US-W	Wyoming	MM	1997	7	7	0	0	0	100.0	0.0
US-W	Wyoming	MM	1998	27	26	1	0	0	96.3	3.7
Can-O	Northwest Territories	MM	1998	51	47	2	2	0	92.2	7.8
Can-A	Alberta	RM	1998	127	123	1	3	0	96.9	3.1
US-A	Oklahoma or Kansas	unknown	1999	142	122	11	6	3	85.9	14.1
Can-C	Saskatchewan	RM	1999	99	88	6	4	1	88.9	11.1
US-W	Wyoming	MM	1999	15	12	3	0	0	80.0	20.0
US-F	Utah	MM	1999	30	30	0	0	0	100	0
US-B	Missouri	unknown	1999	6	2	1	1	2	33.3	66.7
US-C	Nebraska	unknown	1999	294	266	17	10	1	90.5	9.5
Can-M	Alberta	MM	2000	105	105	0	0	0	100	0
Can-P	Northwest Territories	MM	2000	70	66	2	1	1	94.3	5.7
US-W	Wyoming	MM	2000	12	12	0	0	0	100.0	0.0
Can-E	Alberta	RM	2000	149	139	6	3	1	93.3	6.7
Can-F	Alberta	RM	2000	36	29	5	2	0	80.6	19.4
Can-G	Alberta	RM	2000	291	259	20	10	2	89.0	11.0
Can-D	Saskatchewan	RM	2001	150	140	3	2	5	93.3	6.7
US-W	Wyoming	MM	2001	12	12	0	0	0	100.0	0.0
Can-O	Northwest Territories	MM	2001	82	75	4	1	2	91.5	8.5
US-H	New Mexico/Colorado	MM	2001	14	13	1	0	0	92.9	7.1
US-I	Montana	MM	2001	12	12	0	0	0	100	0
US-J	New Mexico	MM	2001	12	12	0	0	0	100	0
US-W	Wyoming	MM	2002	12	11	1	0	0	91.7	8.3
US-W	Wyoming	MM	2003	12	12	0	0	0	100.0	0.0
US-W	Wyoming	MM	2004	12	12	0	0	0	100.0	0.0
US-W	Wyoming	MM	2005	12	12	0	0	0	100.0	0.0
US-W	Wyoming	MM	2009	12	11	1	0	0	91.7	8.3
US-G	Wyoming/Montana	MM	2009	16	16	0	0	0	100	0
US-W	Wyoming	MM	2010	12	11	0	0	1	91.7	8.3
US-W	Wyoming	MM	2011	12	11	1	0	0	91.7	8.3
Can-Q	Saskatchewan	MM	2011	31	28	1	2	0	90.3	9.7
US-G	Wyoming/Montana	MM	2011	2	2	0	0	0	100	0
US-N	Iowa	MM	2012	25	24	0	1	0	96.0	4.0
US-V	New Mexico	MM	2012	40	39	0	1	0	97.5	2.5
US-R	Iowa	MM	2012	8	7	1	0	0	87.5	12.5
US-W	Wyoming	MM	2013	15	15	0	0	0	100.0	0.0
US-R	Iowa	MM	2013	31	29	1	1	0	93.5	6.5
US-U	Wyoming	MM	2013	59	55	0	3	1	93.2	6.8
US-G	Wyoming/Montana	MM	2014	55	51	3	1	0	92.7	7.3
US-K	Nebraska	MM	2014	33	33	0	0	0	100	0
US-L	Montana	MM	2014	26	24	0	0	2	92.3	7.7
US-M	Oklahoma	MM	2014	30	27	1	1	1	90.0	10.0
US-Q	New Jersey	MM	2014	8	8	0	0	0	100	0
US-N	Iowa	MM	2014	19	19	0	0	0	100	0
US-O	Colorado	MM	2014	11	11	0	0	0	100	0
US-O	Colorado	MM	2014	22	22	0	0	0	100	0
US-S	Tennessee	RM	2014	17	15	2	0	0	88.2	11.8
US-T	Oklahoma	MM	2014	40	34	3	2	1	85.0	15.0
US-G	Wyoming/Montana	MM	2015	45	43	2	0	0	95.6	4.4
US-P	North Dakota	MM	2015	6	6	0	0	0	100	0
US-G	Wyoming/Montana	MM	2016	32	32	0	0	0	100.0	0.0
US-W	Wyoming	MM	2016	15	14	1	0	0	93.3	6.7
US-G	Wyoming/Montana	MM	2017	75	71	2	2	0	94.7	5.3
US-W	Wyoming	MM	2017	12	11	0	1	0	91.7	8.3
Can-R	Alberta	MM	2017	33	29	0	3	1	87.9	12.1
US-G	Wyoming/Montana	MM	2018	23	22	1	0	0	95.7	4.3
US-W	Wyoming	MM	2018	12	12	0	0	0	100.0	0.0
US-O	Colorado	MM	2019	12	11	0	1	0	91.7	8.3
US-N	Iowa	MM	2019	9	8	1	0	0	88.9	11.1
Total				3295	3068	126	73	28		

*US* United States, *Can* Canada, *MM* minimally managed animals, *RM* ranch-managed animals

^a^Letters are used to anonymize the specific ranch, herd or other site within the country indicated

^b^Records are unclear as to the specific year of collection

Table 2 summarizes ELISA scores with results delineated according to the country in which samples were collected. Seroprevalence among individual animals in the United States and Canada over the past 36 years is similar, 7.1% (112/1587) and 6.7% (115/1708), respectively, and there is no statistically significant difference in average ELISA scores for sera from these two countries (0.134 and 0.128, respectively; *P* = 0.79). The proportion of seropositive animals in each country for which antibody levels were scored as 2+ or higher is also similar, 42.0% of all positives (47/112) for the United States and 47.0% of all positives (54/115) for Canada, and average ELISA scores for these groups are not significantly different (3.128 and 2.926, respectively, *P* = 0.37).

**Table 2:** Summary of ELISA scores sorted by country of origin

ELISA score	No. (%) of sera collected in the country indicated		Total no. (%)^a^
	United States^a^	Canada^a^	
0 (Negative)	1475 (92.9)	1593 (93.3)	3068 (93.1)
1+ (Weakly positive)	65 (4.1)	61 (3.6)	126 (3.8)
2+/3+ (Moderately positive)	32 (2.0)	41 (2.4)	73 (2.2)
4+/5+ (Strongly positive)	15 (0.9)	13 (0.8)	28 (0.8)
Total	1587	1708	3295

^a^As a result of rounding individual percentages to the nearest tenth, the sum of all percentages does not equal 100

**Fig. 1:**
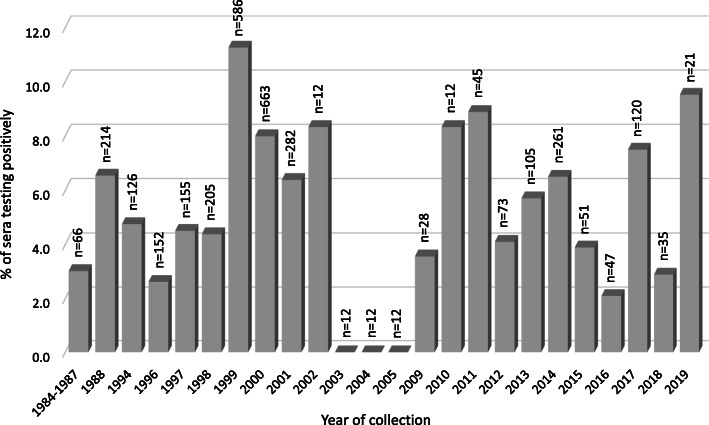
The proportion of bison sera that tested positively with the BIO K260 ELISA, sorted by year of collection. The total number of sera representing each year (n) is given above the corresponding bar

Figure 1 is a graphical summary of the percentage of sera testing positively for each of the years represented in this study. Seropositive bison were identified throughout the entirety of the timeframe covered, beginning in the 1980s, when both weakly positive as well as moderately to strongly positive animals were found in both the United States and Canada (Table 1). The 3 years for which all bison tested negatively (2003–2005) are each represented by only 12 samples, all obtained from herd US-W (Table 1). Bison from herd US-W tested positively when sampled on other occasions, both prior to 2003 and after 2005.

To compare the seroprevalence of *M. bovis* before and after the time that related disease spread throughout North American bison, results from samples obtained in the year 1999 and prior were compared with those from sera acquired in the year 2000 or later (Table 3). Considering the 1504 sera collected between 1984 and 1999, 108 (7.2%) are positive while 119/1791 samples collected between 2000 and 2019 (6.6%) tested positively. There is no statistically significant difference between average ELISA scores obtained for sera representing these two eras (0.124 for 1999 and prior, 0.134 for 2000 and later; *P* = 0.58). When only positive samples are considered, a slightly higher proportion of those collected since the year 2000 tested moderately or strongly positive, specifically, 56/119 or 47.1%, versus 45/108 or 41.7% of sera from 1999 or prior. While the average ELISA score for positive sera collected in the year 2000 or later (2.042) is also higher than the average score for positive sera obtained in 1999 or prior (1.731), the difference falls just short of statistical significance (*P* = 0.06).

**Table 3:** Summary of ELISA scores sorted by era of collection and country of origin

ELISA score	No. (%) of sera collected in 1999 or prior in the country indicated			No. (%) of sera collected in 2000 or later in the country indicated		
	United States	Canada^a^	Total	United States	Canada	Total
Neg	673 (90.6)	723 (95.0)	1396 (92.8)	802 (95.0)	870 (91.9)	1672 (93.4)
1+	43 (5.8)	20 (2.6)	63 (4.2)	22 (2.6)	41 (4.3)	63 (3.5)
2+/3+	18 (2.4)	17 (2.2)	35 (2.3)	14 (1.7)	24 (2.5)	38 (2.1)
4+/5+	9 (1.2)	1 (0.1)	10 (0.7)	6 (0.7)	12 (1.3)	18 (1.0)
Total	743	761	1504	844	947	1791

^a^As a result of rounding individual percentages to the nearest tenth, the sum of all percentages does not equal 100

A comparison of the data obtained for each era that additionally considers the country of origin reveals that bison in the United States sampled in the 1980s and 1990s were more likely to have antibodies to *M. bovis* than bison from Canada sampled during the same timeframe (Table 3), with 9.4% positive (70/743) and 5.0% positive (38/761), respectively. The average ELISA score for all sera collected during the pre-disease era in the United States is double the score for all Canadian samples from that era (0.168 and 0.082, respectively; *P* = 0.002), although the average scores for only those sera testing positively from each country do not differ significantly (1.786 for the United States and 1.658 for Canada; *P* = 0.57). In contrast, 5.0% of sera collected in the United States during the year 2000 and afterwards tested positively (42/844), as compared to 8.1% of Canadian sera (77/947; Table 3). The average ELISA score for Canadian sera obtained during this era, 0.160, is significantly higher than the average for samples collected in the United States during the same timeframe, 0.103 (*P* = 0.05). As seen in the pre-disease era, the average ELISA score for positive sera from the United States does not differ significantly from the average for positive Canadian sera (2.071 for the United States and 2.013 for Canada; *P* = 0.82).

Examination of era-specific data for each country reveals that in both the United States and Canada average ELISA scores for sera collected in 1999 or prior differ significantly in comparison to scores for sera collected afterwards in the same country. However, the direction of change is different in each country. As noted above, average seroprevalence in the United States is 9.4% for samples collected between 1985 and 1999 but only 5.0% for samples collected afterwards. This decrease is accompanied by a significant decrease in average ELISA scores, 0.168 and 0.103 respectively (*P* = 0.03). Average ELISA scores for sera collected in Canada, where average seroprevalence is 5.0% during the pre-disease era and 8.1% in the years following, increased from 0.082 to 0.165, respectively (*P* = 0.003).

### Sera additionally parsed by management practices

To examine whether there is an association between antibody status and management practices, ELISA data arising from minimally managed (MM) bison were compared with data from more intensively reared, ranch-managed (RM) bison (see the Methods section for more details). Relevant information is available for 2631 sera representing 41 unique sources, 23 in the United States and 18 in Canada (Table 1). The percentage of sera from MM bison that tested positively, 5.1% (74/1462), is lower than the percentage from RM bison, 7.4% (87/1169), but the difference in average ELISA scores (0.103 and 0.137, respectively) fails to reach statistical significance (*P* = 0.12). However, it should be noted that only 17/1169 samples collected from RM bison (1.5%) were obtained in the United States, all in the year 2014 (Table 1). Sera from bison in the United States collected in 1999 or prior, when American bison were more likely to test positively, are not represented in the RM group. Because the preponderance of samples from RM animals is from Canada, an additional comparison between MM and MR bison was undertaken using only the data arising from Canadian sera (Table 4). While a lower proportion of sera from Canadian MM bison tested positively, 5.4% (30/556) versus 7.4% (85/1152) for Canadian RM bison, the difference in average ELISA scores for these two groups, 0.108 and 0.137, respectively, is not statistically significant (*P* = 0.32). Nor is any convincing association between management practices and antibody status apparent when results from MM bison are compared with those from RM bison for sera collected during a specific era. The proportion of samples obtained in Canada during the pre-disease era (1999 or prior) that tested positively is nearly identical for MM and RM bison, 5.1% (12/235) and 4.9% (26/526), respectively, as are average ELISA scores for these groups, 0.081 and 0.084, respectively (*P* = 0.93). Considering Canadian samples collected in the year 2000 or later, the prevalence of antibody to *M. bovis* is lowest in MM bison, with 5.6% (18/321) of sera testing positively, versus 9.4% (59/626) of sera from RM bison. Nonetheless, average ELISA scores for these two groups, 0.128 and 0.182, respectively, are not significantly different (*P* = 0.24). However, seroprevalence among Canadian RM bison sampled in the year 2000 or later, 9.4% (59/626 positive), is higher than for RM bison sampled prior to that time, 4.9% (26/526 positive), and the increase is accompanied by a significant rise in average ELISA scores (0.084 in 1999 or prior and 0.182 in 2000 or later; *P* = 0.005). In contrast, seroprevalence in Canadian MM bison during the pre-disease era, 5.1% (12/235 positive), is similar to prevalence in the years 2000–2017, 5.6% (18/321 positive), and the difference in average ELISA scores, 0.081 and 0.128, respectively, is not significant (*P* = 0.31).

**Table 4:** Summary of ELISA scores for Canadian sera sorted by management practices and era of collection

ELISA score	No. (%) of sera collected from MM bison in the era indicated			No. (%) of sera collected from RM bison in the era indicated		
	1999 or prior	2000 or later	Total	1999 or prior^a^	2000 or later	Total
Neg	223 (94.9)	303 (94.4)	526 (94.6)	500 (95.1)	567 (90.6)	1067 (92.6)
1+	7 (3.0)	7 (2.2)	14 (2.5)	13 (2.5)	34 (5.4)	47 (4.1)
2+/3+	5 (2.1)	7 (2.2)	12 (2.2)	12 (2.3)	17 (2.7)	29 (2.5)
4+/5+	0	4 (1.2)	4 (0.7)	1 (0.2)	8 (1.3)	9 (0.8)
Total	235	321	556	526	626	1152

^a^As a result of rounding individual percentages to the nearest tenth, the sum of all percentages does not equal 100

*MM* minimally managed, *RM* ranch-managed

To determine whether there is an association between management practices and the level of antibody found in positive samples, we additionally compared results from positive Canadian MM bison with those from Canadian RM bison. The proportion of positive samples testing moderately to strongly positive (2+ or higher) is similar for MM and RM bison, 53.3% (16/30) and 44.7% (38/85), respectively (Table 4), and average ELISA scores for positive samples from each group are nearly identical, 2.000 and 1.859, respectively (*P* = 0.59). Further segregating data from positive samples based on the time period during which they were collected (1999 or prior and 2000 or later) similarly provides no evidence for an association between the level of positivity and management practices during a particular era, although relatively few sera are available for this analysis. Regarding sera dating to the years 1984–1999, 41.7% of positive samples from MM bison (5/12) and 50% from RM bison (13/26) have ELISA scores ≥2+, with averages of 1.583 and 1.692, respectively (*P* = 0.73). Average scores for positive sera collected in the year 2000 or later are 2.278 for MM bison and 1.932 for RM bison (*P* = 0.35), with 61.1% of MM sera (11/18) and 42.4% of RM sera (25/59) testing at a level of 2+ or greater. The average ELISA scores for positive samples obtained from Canadian MM bison in 1999 or prior do not differ significantly in comparison to those collected from MM bison in 2000 or later (1.583 and 2.278, respectively; *P* = 0.15) and the same holds true for positive sera from Canadian RM bison collected during those two eras (1.692 and 1.932, respectively; *P* = 0.41).

### Herds sampled on multiple occasions

Two Canadian herds, Can-O and Can-P, and five from the United States, US-G, US-N, US-O, US-R and US-W, were sampled on more than one occasion, although only Can-O, Can-P and US-W were sampled during both the pre-disease era and afterwards (Table 1). Seroprevalence in Can-O and Can-P prior to the year 2000 is only slightly less than in the years during which samples were collected afterwards (Table 5). However, in each case the timeframe covered is relatively short, only 3–6 years. In both herds average ELISA scores rose in the years after 1999 but neither increase is statistically significant (*P* ≥ 0.43). Herd US-W was sampled more extensively, in 16 different years over a 22-year period (Table 1). Although seroprevalence in this herd during the pre-disease era is more than double the prevalence found during the years afterwards (Table 5) there is no significant difference in average ELISA scores for each time period (0.082 and 0.068, respectively; *P* = 0.83).

**Table 5:** Summary of ELISA scores for herds sampled both before and during/after the year 2000

Herd	Year	No. of sera	ELISA score					
			0	1+	2+/3+	4+/5+	% pos	Avg ELISA score
Can-O	1998	51	47	2	2	0	7.8	0.118
	2001	82	75	4	1	2	8.5	0.195
Can-P	1994	126	120	4	2	0	4.8	0.071
	2000	70	66	2	1	1	5.7	0.129
US-W	1997–1999	49	45	4	0	0	8.2	0.082
	2000–2018	162	156	4	1	1	3.7	0.068

## Discussion

The data obtained in this study provide novel insights as to the history of *M. bovis* in bison, about which little was known prior to this time. Because it appears the bacterium may have circulated among bison well in advance of its first recognition as a disease problem, a “host jumping” event occurring around that time is unlikely to account for the emergence of disease. Our data, nonetheless, are consistent with the theory that recently evolved, novel genotypes of *M. bovis* may underlie or may have contributed significantly to the appearance of related disease. The abundance of insertion sequences, integrative conjugative elements and other mobile genetic elements found in *M. bovis* leads to considerable genome plasticity as a result of rearrangements, duplications and gene disruptions that can affect the biology of the bacterium through alterations in gene content and expression [13, 14]. These elements may also play a role in horizontal gene transfer, both within and between different *Mycoplasma* species [15], thereby facilitating the emergence of novel variants with unique clinical manifestations. Several instances of newly emergent clones of *M. bovis* associated with specific, novel phenotypes have been reported. Bürki et al. [16] documented the appearance in western Europe of a new lineage of *M. bovis* responsible for severe outbreaks of mastitis in cattle, which had not previously been seen in that region. In an analysis of French cattle isolates collected between 1977 and 2012 [17], three different typing methods each indicated a shift in isolate genotype beginning around the year 2000 that was accompanied by the acquisition of antimicrobial resistance. Unfortunately, we have been unable to acquire bison isolates collected prior to the year 2007, precluding a comparison of genotypes before and after the spread of mycoplasmosis. In the first studies to report genetic characterization of North American bison isolates [12, 18], 60/96 (62.4%) collected in the year 2007 or later were shown to have MLST sequence types (STs) found exclusively in bison. In contrast, a more recent analysis using a revised MLST reference scheme with improved discriminatory power found that only 28.6% of isolates (32/112) have STs unique to bison ([19], https://pubmlst.org/bigsdb?db=pubmlst_mbovis_isolates]). The latter study includes 59 isolates from the previously typed group and 53 additional isolates collected between 2011 and 2015. While MLST can be informative in reconstructing historical phylogenetic relationships, its dependence on nucleotide sequences from housekeeping genes overlooks potential host-restricted evolution and diversity for those genes more directly involved in virulence. Thus, a fuller understanding of the molecular events that andmay have contributed to the emergence of mycoplasmosis in bison requires more extensive genetic characterization of bison isolates. To this end, we have recently sequenced the genomes of a total of 82 bison isolates, 55 from the United States and 27 from Canada, that collectively represent all STs known at this time to infect bison [20, 21]. Comprehensive genome-wide analyses, including comparison with data available for cattle isolates, are currently underway.

It is well-known that *M. bovis* can be carried in the upper respiratory tract of healthy cattle and, on some occasions, even the lower respiratory tract [4]. Carriers sometimes seroconvert while others fail to do so and there is generally no correlation between antibody titers and the development of disease on an individual animal basis [4]. Little information is available with respect to carriage of *M. bovis* in healthy bison. In a prior study [9], we isolated the bacterium from the nasal cavities of 7/8 healthy bison sampled. Data reported here provide additional evidence to suggest that healthy bison may be carriers of *M. bovis*. We cannot discount the possibility that some bison appearing healthy at the time sera were collected may have suffered a prior, unobserved episode of mycoplasmosis. Furthermore, bison have evolved to mask signs of weakness or disease that might attract the attention of predators [22]. However, it seems unlikely that the 227 seropositive bison, or even the 101 that tested moderately to strongly seropositive, might all have experienced active disease without any outward signs being observed by herd managers or other caretakers. Accordingly, our results suggest that at least some bison carriers of *M. bovis* may seroconvert. It must be noted that the presence of antibodies reactive with *M. bovis* in healthy bison serves as only an indirect indicator of carriage or prior infection. Despite exhaustive inquiries, we were unable to acquire archived tissues or other materials collected prior to 2014 that are suitable to attempt either bacterial isolation or the detection of *M. bovis*-specific nucleic acids. A culture-based study currently underway will more definitively establish the present-day incidence and distribution of *M. bovis* carriage in healthy North American bison.

As detailed in the Methods section, the ELISA used here has not been fully validated as a diagnostic test for bison. Whether the cutoff value defined for cattle sera is appropriate for bison sera is presently under investigation. Furthermore, the kit manufacturer makes no claim regarding specificity such that cross-reactivity with antibodies elicited by other species of *Mycoplasma* that infect bison cannot be ruled out (A. Ginter, personal communication). Therefore, our results must be interpreted with caution, particularly for samples testing only weakly (1+) positive. Based on preliminary data, we have thus far found high concordance between results for sera testing ≥2+ with the BIO K260 ELISA and results obtained from Western blots prepared with whole-cell lysates of *M. bovis* (K.B. Register, unpublished observations). Moreover, six of the seven healthy carriers identified in the earlier study mentioned above [9] tested either 1+ (*n* = 4) or 2+ (*n* = 2) positive when their sera were evaluated with the BIO K260 ELISA, and the single, culture-negative bison tested negatively (K.B. Register, unpublished observations). These data, while limited, provide some degree of confidence in the major conclusions of the study reported here, especially considering that sera testing moderately to strongly positive were found in both the United States and Canada during the entire continuum of years represented. Additionally, we noted that 57/101 (56.4%) sera characterized here as moderately to strongly positive have an ELISA score of 3+ or higher. In a prior assessment of various ELISA methods [23], a custom assay utilizing capture antigen prepared from bison isolates performed optimally in comparison to commercially available kits developed for use with cattle sera. The BIO K260 kit used here was among those evaluated but we found the difference in performance as compared to the custom ELISA to be nominal. It was not feasible to use the custom assay for the study reported here, due to the large number of sera tested and the time and labor required to produce a sufficient quantity of antigen. However, data reported here identify a subset of samples for which additional testing with the custom ELISA may more fully reveal the performance characteristics of the BIO K260 ELISA when used with bison sera, which could be of value in ongoing efforts to maximize sensitivity and specificity.

An additional caveat of this study, arising from the decision to include every serum available to us, is that the number of bison sampled on a single occasion from a single herd or site varies widely, from 2 to 294 (Table 1), without any knowledge of the proportion of animals represented by the sera collected. Consequently, data may be skewed, leading to erroneous conclusions, if most positive results arise from a relatively small number of herds with high rates of exposure to *M. bovis* that were disproportionately sampled. In counterpoint, seropositive bison were identified in 78% of the herds represented (32/41, with 16 each in the United States and Canada; see Table 1), of which 68.3% (28/41, with 14 each in the United States and Canada) include bison testing moderately to strongly positive. While this study lacks the rigor of one designed prospectively, with random, proportional sampling, the information gleaned from the samples available nonetheless provides novel insights worthy of further investigation.

## Conclusions

A major conclusion from this study is that North American bison were likely exposed to *M. bovis* many years prior to the emergence of *M. bovis*-related disease. Seroprevalence averaged over the past 36 years is similar in the United States as compared to Canada, but geographic differences are evident when data from each country are further parsed by the era of collection (pre-disease versus post-disease, using the year 2000 as the dividing point). Seroprevalence in the United States during the pre-disease era was significantly higher than in Canada, but was significantly lower than in Canada during the years 2000–2019. In the United States, seroprevalence since the year 2000 has dropped significantly in comparison to seroprevalence during the years 1985–1999, while in Canada seroprevalence has increased significantly since the year 2000, as compared to the 16 years prior. For both eras, the level of antibody detected in positive samples from the United States is no different than the level in positive samples from Canada. Our data further suggest that *M. bovis* may colonize healthy bison in a manner sufficient to trigger antibody responses but without causing overt disease. These findings provide novel insights as to the history of *M. bovis* in bison and will be of value in formulating strategies to better control the spread and minimize the impact of *M. bovis* on bison health and production.

## Methods

The BIO K260 *M. bovis* ELISA (Bio-X Diagnostics, Rochefort, Belgium), designed for use with cattle sera, was used to detect the presence of serum antibodies reactive with *M. bovis*. It was chosen in favor of an alternative product available from the same manufacturer (BIO K302 *M. bovis* ELISA) after consultation with the company’s General Manager and Research & Development Department about the goals of our investigation (A. Ginter, personal communication). Although not fully validated as a diagnostic assay for bison, a prior study demonstrated results to be highly concordant with those from an in-house ELISA developed specifically for bison sera and in agreement with the exposure history of the animals from which the sera were obtained [23]. The sole alternative ELISA commercially available at the time our study commenced utilizes an anti-bovine IgG that reacts poorly with bison IgG [23]. In contrast, the BIO K260 and BIO K302 detect serum antibodies using protein G, which binds with high affinity to IgG of both cattle and bison [24]. All sera were tested on two different occasions, using independently made dilutions, as described in the manufacturer’s instructions. Test values were used to assign each sample a numerical score, from 0 (negative) to 5+, using the following scoring metric provided with the ELISA kit: 0 = a value of ≤37, 1+ = a value > 37 and ≤ 60, 2+ = a value > 60 and ≤ 83, 3+ = a value of > 83 and ≤ 106, 4+ = a value of > 106 and ≤ 129 and 5+ = a value > 129. Sera were recorded as positive only if a result of 1+ or higher was obtained on both occasions tested. Positive sera for which replicate tests yielded discrepant scores (e.g., 1+ versus 2+) were classified based on the average numerical value of both tests. In all such cases the values obtained in each test were similar but were at the boundaries of the cutoff values used to define different levels of positivity. Student’s two-tailed t test was used to evaluate the statistical significance of differences in ELISA scores obtained for the groups of sera indicated, with a *P* value ≤0.05 considered to be statistically significant.

A total of 3295 serum samples from bison in the United States (*n* = 1587) or Canada (*n* = 1708), collected from 1984 through 2019, was available for testing. Some bison were bled twice, in different years, such that the sera collectively represent 3173 bison, with 1548 from the United States and 1625 from Canada. All bison appeared healthy at the time blood was collected. Sera were stored frozen until use, at either − 20 °C or − 80 °C, depending on the institution from which they were obtained. Figure 1 indicates the number of sera collected during each of the years represented in this study. Eight sera in the group with a collection date of 1984–1987 were obtained in 1985; information available for the remaining 58 does not specify the exact year of collection. In some cases, the collection date was recorded as a range of several months spanning the end of 1 year and extending into the next. For those sera, the year assigned as the collection date for purposes of this study is the year in which the majority of the time period falls.

The sera collectively represent a minimum of 23 farms, herds or locations in the United States, mostly in Plains or Western states, and 18 in Canada, mostly from Alberta or The Northwest Territories. For locations Can-A and Can-D (an abattoir and auction market, respectively) no information is available regarding the ranch of origin for individual bison. In each of these instances related sera were considered to have come from a single origin. At 23 of the 36 sites of origin for which information is available regarding rearing practices bison were wide-ranging and minimally managed, with roundups or handling occurring no more than once yearly (1462 samples from 1340 bison). At the remaining 13 sites bison were being more intensively raised at the time of sample collection, with movement restricted by fencing and more frequent handling (1169 samples each representing a single bison). In some instances bison in both categories may have had close contact with cattle.

## Data Availability

The datasets used and analyzed for this study are available from the corresponding author upon request.

## Abbreviations

ELISA: Enzyme-linked immunosorbent assay
MLST: Multilocus sequence typing
MM: Minimally managed
RM: Ranch-managed
ST: Sequence type
